# Risks and benefits of CT angiography in spontaneous intracerebral hemorrhage

**DOI:** 10.1007/s00701-014-2019-7

**Published:** 2014-03-07

**Authors:** Kazuko Hotta, Takatoshi Sorimachi, Takahiro Osada, Tanefumi Baba, Go Inoue, Hideki Atsumi, Hideo Ishizaka, Minako Matsuda, Naokazu Hayashi, Mitsunori Matsumae

**Affiliations:** Department of Neurosurgery, Tokai University School of Medicine, Shimokasuya 143, Isehara City, Kanagawa 259-1193 Japan

**Keywords:** Computed tomography angiography, Intracerebral hemorrhage, Side effect, Spot sign

## Abstract

**Background:**

Few studies have examined the risk of computed tomography angiography (CTA) during the acute phase of spontaneous intracerebral hemorrhage (ICH), while the benefits of CTA in ICH have been well-documented. The present study investigated both the benefits of identifying spot signs, which are supposed to indicate hematoma enlargement after admission, and risks of CTA performed during the acute phase of ICH.

**Methods:**

We retrospectively assessed 323 consecutive patients with spontaneous ICHs admitted to our hospital between April 2009 and March 2012 and who underwent CTA on admission.

**Results:**

In 80 patients (24.7 %), spot signs were demonstrated on CTA source images. Multivariate analysis revealed two independent factors correlated with presence of the spot sign: age and hematoma volume (*p* < 0.05 each). The presence of spot sign was associated with unfavorable outcomes at discharge and hematoma growth after admission (*p* < 0.05 each). Adverse events related to CTA occurred in 17 patients (5.2 %), including transient renal dysfunction in 16 patients and allergy to contrast medium in one patient. All adverse events completely resolved within 1 week.

**Conclusions:**

Presence of the spot sign indicated the possibility of hematoma growth and unfavorable outcomes. A small number of adverse events occurred in association with CTA, but without any permanent deficits. Given the potential benefits and risks, we believe that CTA performed at admission in all patients with ICH is beneficial to improve the outcomes.

## Introduction

Intracerebral hemorrhage (ICH) has a more unfavorable outcome than ischemic stroke in terms of the grade of poststroke disability and mortality rate [1, 4]. Hematoma expansion has been identified as one of the most important determinants of early neurological deterioration and unfavorable clinical outcome [5, 6]. The spot sign is a high-density area of contrast media leakage within the hematoma on a source image from computed tomography angiography (CTA) performed in the acute stage of ICH. Several studies have reported that the presence of spot sign predicts hematoma growth [7, 8, 14]. Patients with the spot sign may be candidates for management with intensive blood pressure (BP)-lowering or hemostatic therapies. Although CTA has possible side effects due to the bolus injection of contrast media, such as on renal function and/or allergy to contrast medium, few studies have reported on the risks of CTA during acute-stage ICH [13, 16, 22].

The present study first investigated predictive factors for occurrence of the spot sign in patients with ICH undertaking CTA immediately after admission. We then evaluated relationships between the spot sign and outcomes. Finally, we examined the risk of CTA performed immediately after the onset of ICH.

## Methods

### Patient population

We retrospectively assessed consecutive patients with spontaneous ICH admitted to our hospital between April 2009 and March 2012. Patients admitted later than 24 h after onset were excluded. This series of patients did not include those with secondary ICH caused by cerebral aneurysm, vascular malformation, moyamoya disease or trauma. Patients who did not undertake CTA on admission were excluded. Patients presenting with hemorrhagic stroke admitted to our neurosurgical unit routinely underwent the first non-contrast CT on arrival, followed by a CTA study, unless a history of renal dysfunction and/or allergic history to contrast agent was obtained. Emergency hematoma removal was performed in patients less than 80 years old with severe disturbance of consciousness due to mass effect of the hematoma or with impending tentorial herniation due to the hematoma. Ventricular drainage was performed in patients with acute hydrocephalus on non-contrast CT. The study was approved by the ethics committee at Tokai University School of Medicine.

### Image acquisition

The CT protocol was performed on a 40 slice CT (Brilliance; PHILIPS, Best, the Netherlands). Pre-contrast head imaging was acquired from the skull base to the vertex under the following parameters: collimation, 40 × 0.625 mm; 120 kVp; 450 mA; rotation time, 1 s/rotation tube, at table speed 15 mm/rotation. CTA was performed from the C4 cervical level to the vertex under the following parameters: collimation, 40 × 0.625 mm; 120 kVp; 370 mA; rotation time, 0.5 s/rotation; table speed, 15 mm/rotation. The CTA protocol was performed using a bolus tracking method in which non-ionic contrast agent (iohexol or iopamidol) was injected at 4 ml/s through a peripheral vein via an indwelling 20-G angiocatheter. Pre-contrast images were reconstituted as axial, 5-mm-thick images. Three-dimensional CTA images were reconstituted as axial and coronal, 3-mm-thick images. All images were viewed on the software of a computer terminal.

### Image analysis

All studies were evaluated by 10 neurosurgeons separately for the presence or absence of spot signs by simultaneously visualizing non-contrast CT studies cross-linked with coronal axial CTA reformats. Spot sign was defined as an enhancing focus >1 mm in diameter within a hematoma on a CTA source image. Maximum attenuation was defined as more than twice the Hounsfield unit (HU) value of the surrounding hematoma, or >120 HU [24]. A consensus reading by several neurosurgeons was conducted to resolve any ambiguous findings regarding the interpretation of CTA source images. Hematoma volume was calculated using the ABC/2 method [15]. Pre-contrast CT was performed on admission and on the second day after admission. Hematoma growth was defined as a volume increase of ≥12.5 ml or ≥33% [24].

### Data collection

Clinical data were obtained through chart review. The following data were recorded: patient age and sex, level of consciousness (Glasgow coma scale [GCS] score) on admission, systolic BP on admission, use of antiplatelet agents, use of anticoagulants, and time from ICH onset to admission. Clinical outcomes were assessed using the modified Rankin Scale (mRS) score at discharge. Favorable and unfavorable outcomes were defined as mRS 0–2 and 3–6, respectively.

Acute renal dysfunction, allergic symptoms, and anaphylactic reactions to contrast medium were investigated as complications caused by CTA. In all patients, serum creatinine (Cr) levels were examined for three consecutive days after admission. Renal dysfunction, based on the definition of the Radiological European Society of Urogenital Radiology [21], was defined as a ≥25% increase over the reference value, or an increase of >0.5 mg/dl in serum creatinine within 3 days of exposure to the contrast media. The occurrence of clinical signs of hypotension, nausea, vomiting, urticaria, or pruritus after CTA was investigated as potential anaphylactic reactions or allergic symptoms.

### Statistical analysis

Univariate analysis was performed using chi-squared analysis and Fisher’s exact probability test for categorical variables and using one-way analysis of variance for continuous variables. Numerical data are expressed as the mean ± standard deviation.

Each variable was analyzed using univariate analysis to identify possible significant predictors; those variables found to be possibly significant at the *p* < 0.10 level were then included in multivariate logistic regression analysis, which was reduced by successively removing the least significant variable from the model. All variables showing a value of *p* < 0.10 were kept in the final model. Analyses resulting in values of *p* < 0.05 were considered statistically significant. All statistical analyses were performed using of JMP version 10 software (SAS Institute, Cary, NC, USA).

## Results

Between April 2009 and March 2013, we treated 568 consecutive patients diagnosed with spontaneous ICH. Of the 568 patients, 15 admitted >24 h after onset and 230 who failed to undergo CTA on admission were excluded. A total of 323 patients met the inclusion criteria of the study, with a mean age of 67.0 ± 13.0 years. Hematomas were present in the putamen in 105 patients (32.5 %), thalamus in 71 (29.2 %), both putamen and thalamus in 12 (4.9 %), cortex in 35 (14.4 %), subcortex in 28 (11.5 %), cerebellum in 35 (14.4 %), brainstem in 12 (4.9 %), and caudate nucleus in 11 (4.5 %). External ventricular drainage or hematoma removal was performed in 12 and 87 patients, respectively.

In 80 of the 323 patients (24.7 %) who underwent CTA, spot signs were demonstrated on CTA source images. Table 1 shows the following factors for patients with and without spot sign: age, sex, use of antiplatelet agents, hematoma location, time from symptom onset to admission, level of consciousness (GCS score) on admission, systolic BP on admission, hematoma volume, and presence of intraventricular hemorrhage. In univariate analyses, significant differences were found in age and hematoma volume between spot sign-positive and -negative groups. Multivariate analysis revealed two independent factors correlated with presence of spot sign: age (*p* = 0.0160; odds ratio [OR], 1.030) and hematoma volume (*p* = 0.0063; OR, 1.006).

**Table 1:** Differences in clinical factors between patients with and without an early spot sign

Variable	Patients with spot signs (*n* = 80)	Patients without spot signs (*n* = 243)	*P* value one-way analysis	OR (95 % CI)	*P* value
				Multivariate analysis	
Age (years)	71.1 ± 12.0	66.2 ± 12.7	0.0014	1.030 (1.009–1.054)	0.0160
Use of antiplatelet drugs	17	38	0.2467		
Male	51	136	0.2214		
Putamen	26	79	0.9986		
Thalamus	20	51	0.4523		
Brain stem	4	8	0.4836		
Cortex	17	58	0.6516		
Caudate head	0	11	0.0717		
Cerebrum	8	27	0.7815		
Time from onset to admission (min)	208.261 ± 705.856	288.919 ± 788.732	0.7747		
GCS < 8 at admission	28	63	0.1176		
SBP at admission (mmHg)	183.986 ± 30.3922	181.539 ± 40.5162	0.3127		
Hematoma volume (cm^3^)	54326.9 ± 47330.3	36583.6 ± 49927.9	0.0028	1.006 (1.001–1.011)	0.0063
Intraventricular hemorrhage	44	117	0.3492		

*GCS* Glasgow Coma Scale; *SBP* systolic blood pressure

**Fig. 1:**
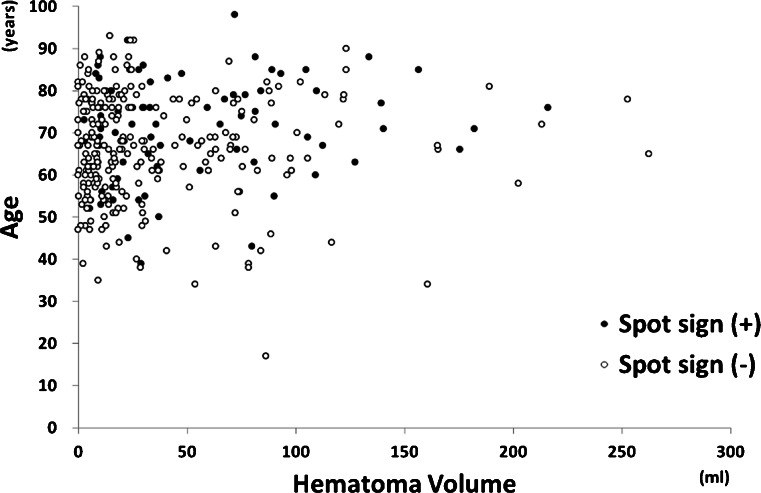
A relationship between age and hematoma volume, which are independent predictors for spot signs, and presence of spot signs. No clear cutoff value of age or hematoma volume for presence of spot signs is demonstrated. A *black circle* indicates a patient with spot signs, and a white circle a patient without spot signs

Figure 1 shows a relationship between age and hematoma volume, which were the independent predictors for spot signs, and presence of spot signs. This graph does not show any apparent cut-off value of age or hematoma volume for presence of spot signs.

Table 2 shows the relationship between presence of spot sign and hematoma growth on CT on the day after onset. Patients, who underwent emergency hematoma removal, comprising 33 patients with spot sign and 54 without spot sign, were excluded, because follow-up CT could not be performed for evaluation of hematoma growth. Hematoma enlargement was found on CT the day after admission in 15 of 40 patients (37.5 %) with spot sign and in 14 of 158 (8.9 %) without spot sign. The frequency of hematoma enlargement was significantly larger in patients with spot sign than in patients without spot sign (*p* < 0.0001).

**Table 2:** Relationships between spot sign occurrence and hematoma enlargement

	Spot sign	
	Positive (*n* = 80)	Negative (*n* = 238)
Hematoma enlargement	15	14
Hematoma stable	25	144
Not available^a^	40	80

^a^Not available = CT was not performed the day following admission because of hematoma removal, death, or other reasons

**Fig. 2:**
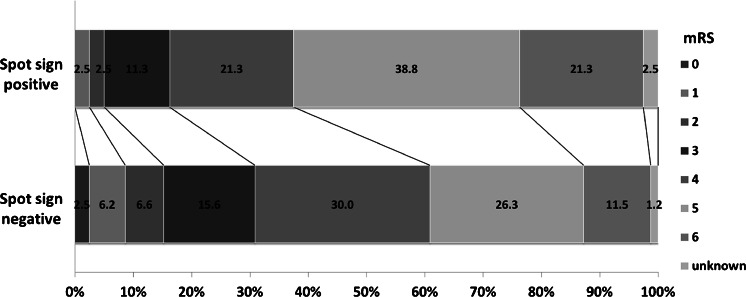
Outcomes for patients with spontaneous intracerebral hemorrhage with or without spot signs. Favorable outcome (mRS 0-2) was significantly less frequent in patients with spot signs than in patients without spot signs

Figure 2 demonstrates the relationship between presence of spot sign and outcome. Favorable outcomes, defined as mRS ≤ 2 at discharge, were obtained four 4 of 80 patients (5.0 %) with spot sign, and 37 of 243 (15.2 %) without spot sign. The frequency of favorable outcomes was significantly smaller in patients with spot sign than those without (*p* = 0.0158). Death occurred in 17 of 80 patients (21.3 %) with spot sign and 28 of 243 patients (11.5 %) without spot sign, and was thus significantly more frequent with the spot sign (*p* = 0.0327). Relationships between spot sign and outcome were re-examined separately in patients with hematoma volume ≥50 ml and in patients with hematoma volume <50 ml, since hematoma volume strongly influences outcome (Table 3). In patients with hematoma volume <50 ml, the frequency of unfavorable outcomes was significantly larger in patients with spot sign than in patients without spot sign (*p* = 0.01149). On the other hand, in the group with hematoma volume ≥50 ml, no significant difference in outcome was apparent between patients with and without spot sign.

**Table 3:** Relationships among hematoma volume, presence of spot sign and outcome

		Hematoma volume			
		<50 ml (*n* = 224) Spot sign		≥50 ml (*n* = 96) Spot sign	
		Positive (*n* = 47)	Negative (*n* = 177)	Positive (*n* = 33)	Negative (*n* = 63)
mRS	0–2	3 (6.4 %)	34 (19.2 %)	1 (3.0 %)	3 (4.7 %)
	3–6	44 (93.6 %)	143 (80.8 %)	32 (97.0 %)	60 (95.2 %)

Three patients, in which the hematoma volume value was not available, were excluded

**Fig. 3:**
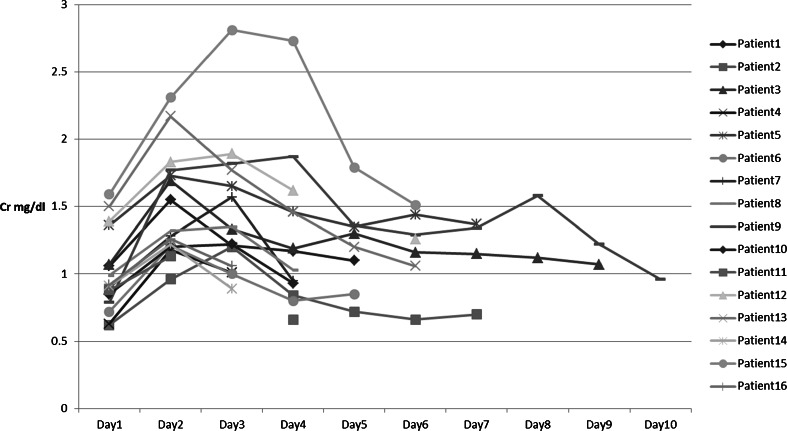
Changes of serum creatinine levels in patients suffering from contrast medium-induced nephropathy. All 12 patients in renal failure after admission show improvement of serum creatinine levels to the previous level within 7 days

Adverse events of CTA occurred in 17 of 323 patients (5.2 %), comprising transient renal dysfunction in 16 patients and allergy to contrast media in 1 patient. No other complications related to CTA were encountered. Figure 3 demonstrates the time course of Cr levels in the 16 patients with renal dysfunction. In all these patients, Cr levels normalized within 1 week without any aggressive treatment, including dialysis. The patient who suffered from allergic reaction to contrast media showed hypotension immediately after administration of the contrast media for less than 10 min. Symptoms improved from 5 h after onset with hydration and administration of antihistamine.

## Discussion

The present study identified patient age and hematoma volume as predictors for occurrence of the spot sign. Progression of arteriosclerotic changes in the perforating arteries, which are etiological arteries for ICH, was considered to result in the more frequent observation of spot sign in older patients compared with the younger patients [9, 23]. Increased vulnerability to rupture of arteries in the arteriosclerotic perforating arteries could prevent hemostasis after the onset of ICH. Large hematoma volume, another predictor of spot sign, might be the result of ongoing bleeding expressed as the spot sign. In previous studies, several factors including hematoma volume, GCS, time from onset to admission, blood glucose level >170 mg/dl, mean BP on admission >120 mmHg, higher prothrombin time (PT)-INR, and use of antiplatelet agents and anticoagulants has been reported as a predictor for spot sign [3, 24]. The present study revealed age as a predictor for spot sign, a finding that has not been reported previously. The large number of patients that participated in this study might have facilitated the detection of this previously unrevealed finding. Compared with Western populations, the Japanese prevalence of ICH might explain the different result between the present study and previous investigations. Our study showed no apparent cut-off value of age or hematoma volume for presence of spot signs. So age and hematoma volume could not be used as indicators to perform CTA.

The significantly larger frequency of hematoma enlargement in the group with positive spot sign demonstrated in this study resembled the results of previous reports [7, 8, 12, 14]. The spot sign was considered to indicate ongoing bleeding from the perforating arteries, which resulted in hematoma enlargement. In consideration of the hematoma expansion observed in patients without spot sign, other possible mechanisms of hematoma expansion may include oozing of blood from arteries and/or veins, which failed to show the spot sign, and intermittent bleeding from arteries.

In patients with hematoma volume <50 ml in this study, the frequency of unfavorable outcomes was significantly higher in patients with spot sign compared to those without spot sign. On the other hand, among those patients in whom hematoma volume was ≥50 ml, no significant differences in outcome were observed between groups with and without spot sign. Hematoma volume is a well-known predictor of unfavorable outcomes, as well as a predictor for the presence of spot sign [7, 8, 14]. We investigated relationships between spot sign and outcome separately in patients with hematoma volume ≥50 ml and <50 ml, because hematoma volume is a possible confounding factor for both spot sign and outcome. In ICH showing both spot sign and hematoma volume <50 ml, therapeutic intervention aimed at preventing hematoma expansion, including strict blood pressure control and hemostatic agents, could be considered.

Several randomized control trials have been recently conducted to clarify an effect of aggressive medical treatment or emergency surgery on outcomes in patients with spontaneous ICHs. The Intensive Blood Pressure Reduction in Acute Cerebral Hemorrhage Trial 2 (INTERACT2), which is a randomized control trial to test an effect of blood pressure lowering on outcomes in ICH patients, showed intensive lowering of blood pressure with a target systolic level of <140 mm Hg within 1 h improved functional outcomes in comparison to American Stroke Association guidelines-recommended treatment with a target systolic level of 180 mm Hg [2]. Two phase II trials of rFVIIa in ICH patients using spot sign selection are underway in Canada and the United states [10, 11], though improvement in survival and functional outcomes by use of rFVIIa were not found in a large phase III randomized control trial without spot sign selection [17, 18]. An examination of spot sign and tranexamic acid on preventing ICH growth, which is a phase II randomized control trial, is also ongoing in Australia [20]. Identification of spot sign in ICH becomes more important if the results of these trials show improvement of outcomes in the aggressive medical treatment group in the future.

Spontaneous supratentorial lobar intracerebral hematomas (STICH II) trial, which was a randomized control trial to investigate an effect of early surgery on outcomes in patients with superficial lobar intracerebral hemorrhage, did not show significant evidence to support the benefit of early surgery [19]. In our study, emergency surgery was performed in patients showing herniation sign to save their lives and not to improve their functional outcomes. We believe that strict blood pressure control, careful observation of the neurological status, and preparation of emergency surgery to save life should be performed during the first 24 h in patients showing spot signs.

The present study demonstrated that adverse events associated with CTA occurred in 5.2 % of patients, consisting of acute renal dysfunction in 4.9 % and allergic reaction in 0.3 %. In all patients, acute renal dysfunction resolved completely within 1 week. No residual impairment related to CTA was shown in this series. The bolus injection of contrast media used for CTA can result in more serious side effects compared with usual contrast CT. In addition, CTA was performed immediately after arrival in the emergency department, so a detailed history of renal function could not be obtained, and laboratory data related to renal function could not be obtained prior to CTA. Studies into the adverse effects of CTA on renal function in the acute stage of ICH are scarce. One such study reported that renal dysfunction occurred transiently, if present [22]. For the prevention of renal dysfunction, infusion of a sufficient volume of fluid should be performed after CTA, especially in the acute stage of ICH. An effort to elicit the history of renal function should be made on admission. According to the attached document of iohexol, hypotension after injection of the contrast medium occurred in 28 of 18,657 cases (0.15 %). The frequency of hypotensive reaction to contrast medium in the present study was similar to the reported rate.

## Conclusions

In this study, spot sign on a CTA source image was demonstrated in 80 of 223 patients (24.6 %) with acute ICH. Predictors for the presence of spot sign were age and initial hematoma volume on CT. In patients with hematoma <50 ml, outcomes were significantly worse in patients with spot sign than in those without. A CTA study of acute ICH cases could elucidate the etiologies of secondary ICH as well as predict outcomes in primary ICH. No permanent complications related to CTA occurred in the acute stage of ICH. We believe that CTA performed at admission in all patients with ICH is beneficial to improve the outcomes in consideration of both the benefit and risk of CTA demonstrated in the present study.
